# Do Total Hip Replacements Improve Outcomes for Patients With Neck of Femur Fractures?

**DOI:** 10.7759/cureus.93199

**Published:** 2025-09-25

**Authors:** Piers A Johal, Soubhik Ghosh, Aniruddh Shinde, Odisseas Shablahidis, Arun Bhaskaran

**Affiliations:** 1 General Medicine, Darent Valley Hospital, London, GBR; 2 Trauma and Orthopaedics, Queen Elizabeth Hospital, London, GBR; 3 Trauma and Orthopaedics, Morriston Hospital, Swansea, GBR

**Keywords:** constrained liner, dual mobility cup, hip fracture outcomes, neck femur fracture, ortho surgery, total hip replacement (thr)

## Abstract

Introduction

Neck of femur (NOF) fractures are a significant challenge in the elderly population, with high morbidity and mortality rates. Total hip replacement (THR) is a well-established treatment for displaced intracapsular NOF fractures. However, THR carries a higher risk of complications such as dislocation and periprosthetic fractures in elderly patients. This study aims to assess the management, complications, and functional outcomes of THR in displaced NOF fractures in the elderly.

Methods

A retrospective review was conducted on patients with displaced intracapsular NOF fractures treated at a major teaching hospital from January 2014 to November 2017. Patient data was collected from the National Hip Fracture Database. Functional outcomes were assessed using the Oxford Hip Questionnaire at least one year postoperatively.

Results

A total of 1,831 patients with NOF fractures were included, with 134 (16.1%) undergoing THR. The mean time to surgery for THR was 1.8 days. Complications included two acetabular periprosthetic fractures, four periprosthetic shaft fractures, and five dislocations. No 30-day mortality was observed, but four patients died within one year, primarily from unrelated causes. The average Oxford Hip Score was 39.8/48, indicating good functional outcomes in most patients.

Conclusions

THR provides favorable functional outcomes for displaced NOF fractures in selected elderly patients, although complications such as periprosthetic fractures and dislocations remain a concern. Careful patient selection is key, as increased mortality was seen in THR for NOF versus THR for osteoarthritis.

## Introduction

Neck of femur (NOF) fractures are a significant concern in the elderly population, with an annual incidence of over 72,000 cases in the UK [1]. These fractures are commonly the result of low-energy falls and are more common in individuals with osteoporosis. Managing NOF fractures poses considerable challenges due to high mortality and morbidity rates, as well as an increasing cost burden. The total cost of care for NOF fractures in the UK exceeds £2 billion annually, with a 30-day mortality rate of 6.7% and a one-year mortality rate of up to 30% [2,3].

Total hip replacement (THR) surgery is a well-established procedure for treating various hip conditions, including osteoarthritis, rheumatoid arthritis, and hip fractures [4]. THR involves replacing the damaged femoral head and acetabulum with prosthetic components, which can be either cemented or uncemented. This surgery aims to relieve pain, restore hip function, and improve the quality of life for patients.

There are several types of prostheses used in THR for displaced intracapsular NOF fractures. Conventional THR, which typically involves a standard femoral stem and acetabular cup, is the most common approach. It offers good long-term outcomes and functional recovery but may carry a risk of dislocation in certain patient populations, such as the elderly or cognitively impaired. In select cases, to enhance joint stability and reduce the risk of dislocation, surgeons may opt for alternative implant designs such as dual mobility (DM) liners or constrained liners. The dual mobility construct incorporates two articulations: one between the femoral head and a mobile polyethylene insert, and another between the insert and the acetabular shell. This design provides a greater range of motion and lower dislocation rates compared to conventional THR, but potential complications include cup loosening, polyethylene wear, and infection. Constrained liners, developed to manage recurrent instability, mechanically capture the femoral head within the acetabular component. While they provide enhanced stability, they can limit range of motion and are associated with higher rates of impingement, polyethylene wear, and mechanical failure over time. The choice of implant is influenced by both patient factors and surgeon preference, particularly in cases where stability is a primary concern.

In the context of displaced intracapsular neck of femur (NOF) fractures in the elderly, total hip replacement (THR) is considered under specific conditions. According to the National Institute for Health and Care Excellence (NICE) clinical guidelines, THR should be offered instead of hemiarthroplasty to patients who were independently mobile outdoors with no more than one walking aid, have no significant cognitive impairment, are expected to be able to carry out activities of daily living independently beyond two years, and are medically fit for surgery [5]. Selection criteria commonly include a pre-injury mobility status of walking independently or with a single stick, cognitive function with an Abbreviated Mental Test Score (AMTS) [6] greater than 8, and an American Society of Anaesthesiologists (ASA) physical status classification of grade I or II [7]. These criteria aim to ensure that the selected patients are likely to benefit from the procedure and achieve a favorable functional outcome.

The advantages of THR over other surgical options, such as hemiarthroplasty or internal fixation, include better restoration of hip function and potentially lower rates of reoperation [8]. However, THR in elderly patients with NOF fractures is associated with higher risks of dislocation, infection, and periprosthetic fractures due to the fragile skeletal structure and comorbidities prevalent in this age group [9]. Furthermore, the expectations and outcomes for THR in trauma cases differ significantly from those in elective surgeries for osteoarthritis, with trauma patients often having a higher complication rate and mortality [10].

Given these complexities, this study aims to assess the management strategies for displaced intracapsular NOF fractures treated with THR, identify complications, and evaluate functional outcomes.

## Materials and methods

A retrospective review was conducted on patients who were admitted with displaced intracapsular NOF fractures over 42 months between January 2014 and November 2017 at the Morriston Hospital, Swansea.

Demographic variables and patient data were collected from the National Hip Fracture Database for the hospital. These were used as part of the preoperative workup prior to THR. ASA status was recorded from operating theater records and preoperative reviews. An AMTS was collected preoperatively. The mobility status of the patient was ascertained via consultation and physical assessment. The timing and type of surgery were also identified.

An internal independent audit of the data collected from the National Hip Fracture database was conducted to correct and confirm any information that was collected regarding ASA status, AMTS, mobility status, timing, and type of surgery.

The Oxford Hip Questionnaire [11] was sent to all patients who were alive at least one year post-surgery, and appropriate permissions were sought to use the instrument. The Oxford Hip Questionnaire is a short 12-item survey that assesses pain and function of the hip in relation to daily activities. Each item is scored on a scale from 0 to 4, with the total score ranging from 0 to 48, where higher scores indicate better hip function and less pain.

The inclusion criteria for the study included patients with a displaced intracapsular hip fracture who were able to walk independently outdoors with no more than the use of a stick, do not have a condition or comorbidity that makes the procedure unsuitable for them, and are expected to be able to carry out activities of daily living independently beyond two years.

The exclusion criteria were patients who were not fit for anesthesia, patients with significant medical comorbidities, patients with dementia, patients with neuromuscular conditions, patients who could not mobilize independently, and patients with a life expectancy of less than two years.

## Results

A total of 1,831 patients who had NOF fractures were assessed in the study. Overall, 1,040 were intracapsular fractures (57%) and 791 were extracapsular (43%). Of the intracapsular fractures, 829 (80%) were displaced, whereas 221 (20%) were non-displaced (Table 1).

**Table 1:** Distribution of 1,831 patients included in the study by fracture type, including subclassification of intracapsular fractures into displaced and non-displaced

Type of fracture	Number of patients
Non-displaced intracapsular	211 (12%)
Displaced intracapsular	829 (45%)
Extracapsular	791 (43%)

**Figure 1:**
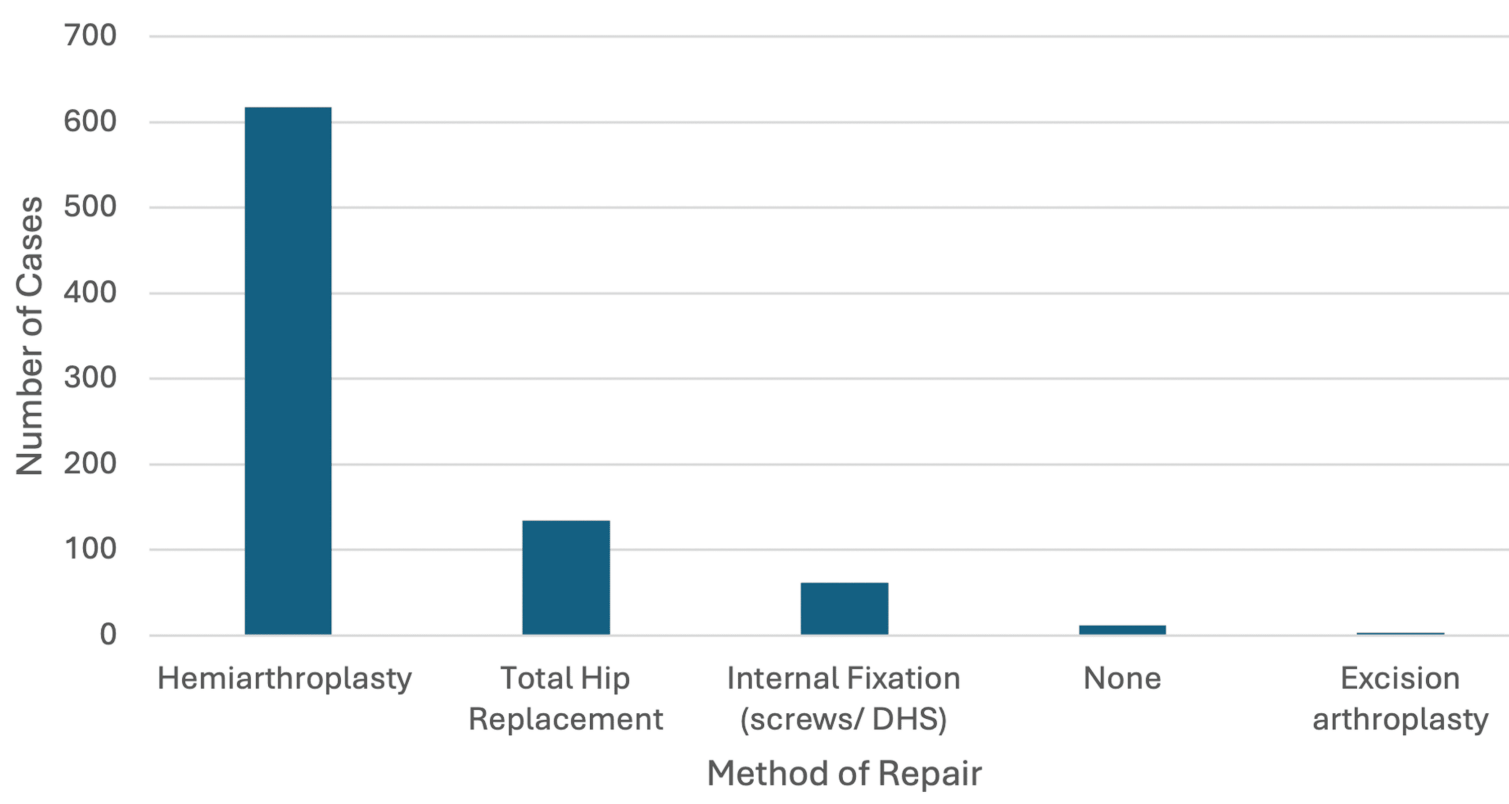
Different methods used to manage the 829 displaced intracapsular neck of femur fractures DHS: dynamic hip screw

Of the 829 displaced intracapsular NOF fractures, 618 (74.5%) underwent a hemiarthroplasty, 134 (16.1%) underwent a total hip replacement, 62 (7.4%) underwent internal fixation with screws or dynamic hip screws, 12 (1.4%) did not undergo surgery, and three underwent excision arthroplasty (Figure 1).

For patients undergoing a hemiarthroplasty, the mean time to surgery was 1.4 days with a range of 0-5 days. For those undergoing a total hip replacement, the mean time to surgery was 1.8 days, and the range was 0-4 days. A common reason for the delay in surgery was a lack of medical optimization; patients receiving anticoagulation such as warfarin or a direct oral anticoagulant needed to be reversed or held for an appropriate period of time. Another reason for the delay in time to surgery was the unavailability of the arthroplasty-trained surgeons. This is significant when THR is required, as THR often requires a specialist hip surgeon, whereas hemiarthroplasty can generally be performed by on-call orthopedic trauma surgeons. The NICE guidelines recommend that surgery be performed within 36 hours of admission to optimize patient outcomes [12].

The eligibility criteria to undergo a THR were an AMTS ≥ 8, being able to mobilize with one stick or better, and being medically and anesthetically fit for the procedure. In the cohort, 305 patients met the eligibility criteria for THR. Overall, 134 patients underwent the procedure, which was 43.9% of those eligible. Of the patients undergoing THRs, 78% were women and 22% were men. All operations were done using a posterior approach to the hip. A total of 110 THRs were hybrid replacements, and 24 were uncemented. Several stem designs were used when performing THRs: cemented stems were taper-slip polished designs, uncemented were hydroxyapatite-coated collarless designs, long uncemented distal stems were used, and a mini hip stem was used in a young patient. A multitude of articulation surfaces were used when performing THRs: 91 were metal on poly, 35 were metal head with dual mobility, two were ceramic on poly, five were ceramic on ceramic, and one was metal on metal. Four different head sizes were used when performing THRs: 35 were dual mobility cups, 67 were 36 mm diameter, 30 were 32 mm diameter, and two were 28 mm diameter.

Some complications were reported. There were two periprosthetic fractures of the acetabulum. One was an uncemented cup with a posterior wall fracture, which was noted postoperatively and underwent cup revision. Post-revision, an infection was also identified in this case, which did not require a return to the theater and was managed with antibiotics. The other case was a perioperative fracture of the posterior wall noted perioperatively, which was stable, and no further input was required.

**Figure 2:**
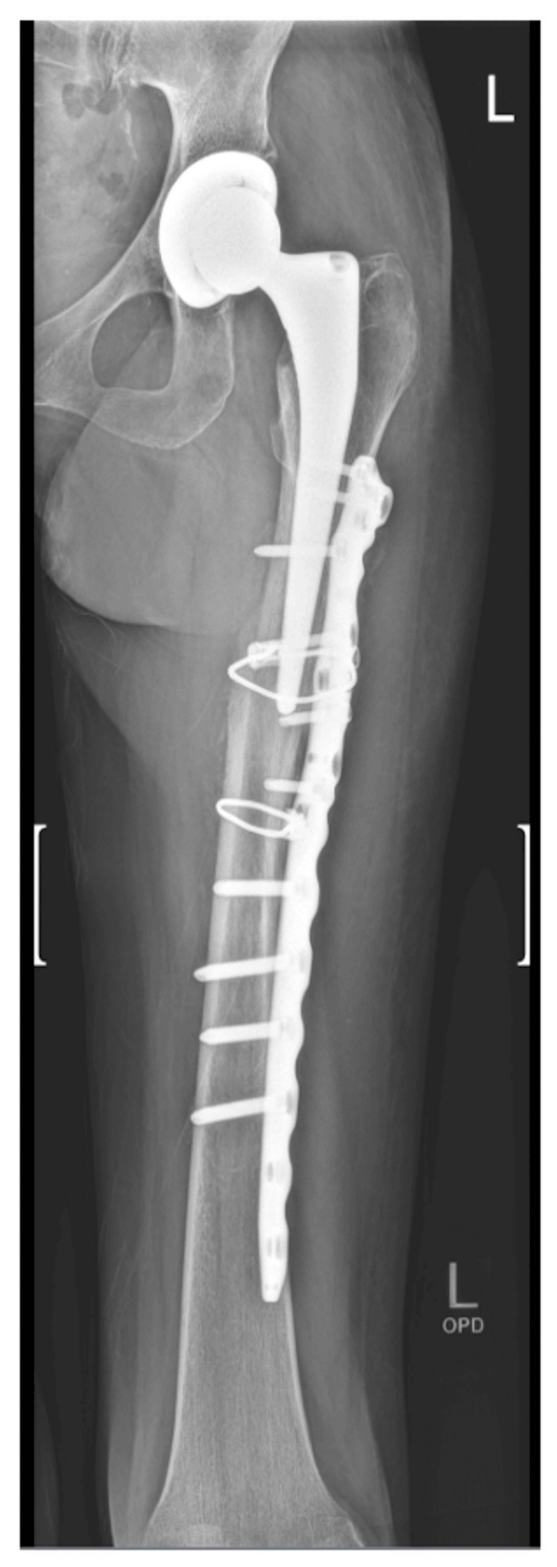
Left femoral anterior-posterior X-ray showing a periprosthetic fracture of the stem in a 54-year-old female patient

**Figure 3:**
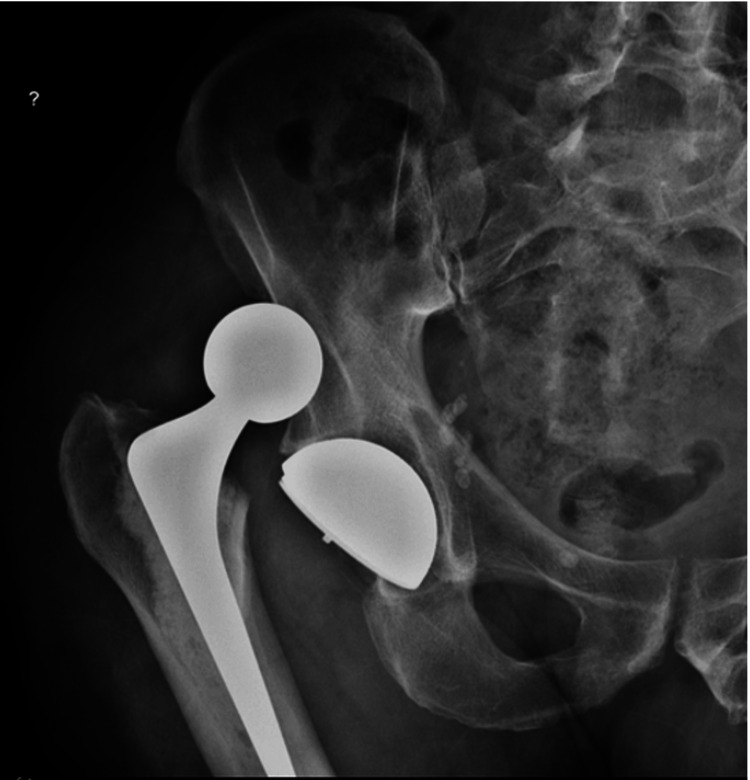
Right pelvic anterior-posterior X-ray showing a dislocation of an implant in a 68-year-old male patient

Other complications included femoral shaft periprosthetic fractures of the stem (Figure 2), resulting from unrelated trauma. Two fractures were for uncemented coated stems within six weeks of THR. Both required open reduction and internal fixation. Two fractures were for cemented taper-slip stems after six months. Both also required open reduction and internal fixation. Dislocation was a complication in five cases: three occurred for the 36 mm heads, two for the 32 mm heads, and none for the DM cups (Figure 3).

With regard to mortality, at 30 days, there was none. At one year, four patients were identified: three were metastatic malignancies and the other one had a head injury following a separate fall. These were excluded from the study.

Functional outcomes were ascertained from the National Hip Fracture Database for the hospital. All clinic letters and hospital notes were also reviewed for revision procedures. A snapshot of the Oxford Hip Questionnaire was posted or discussed over a telephone consultation. Eighteen patients were not able to be contacted, but clinical progress was noted to be good. The average Oxford Hip Score was 39.8/48 from the 109/127 responses collected.

Biasing for low scores was assessed during telephone consultations. Reasons were found to be adjacent joint or spine morbidity, old age, and medical comorbidities such as a stroke.

## Discussion

The results of this study offer key insights into the operative techniques, implant variability, and clinical outcomes associated with total hip replacement (THR) for neck of femur (NOF) fractures.

One area of focus is the choice of implant, particularly the differences between hybrid, cemented, and uncemented prostheses. Hybrid THRs may offer a favorable balance between stability and longevity. Recent data from the National Joint Registry 2024 indicate that cemented stems have the lowest cumulative revision rates among the three types, with the best-performing cemented THRs showing a 2.66% revision rate, closely followed by uncemented (2.70%) and hybrid (2.72%) stems [13]. This supports the view that cemented prostheses offer a stable, reliable option for elderly patients with fragile bone stock.

Although our study observed an equal number of periprosthetic fractures in both cemented and uncemented designs, previous research has suggested that uncemented stems may pose a higher risk of such complications, particularly in elderly patients with fragile bone stock. Supporting this, a study from the Dutch Arthroplasty Register involving 43,053 patients aged 80 and older found that uncemented THRs had higher revision rates compared to cemented THRs. The most frequent reason for revision in uncemented THRs was periprosthetic fracture, accounting for 38% of cases, whereas dislocation was more common in cemented THRs [14]. Additionally, a meta-analysis comparing functional outcomes and complications between cemented and uncemented THRs in elderly patients with NOF fractures reported that cemented THRs had superior Harris Hip Scores both in the short term (<5 years) and long term (>5 years). The study also noted that uncemented THRs had significantly higher complication rates, including radiographic loosening, periprosthetic fractures, dislocation, and heterotopic ossification. Revision rates were also higher in the uncemented group (66/444) compared to the cemented group (27/441) [15]. These findings underscore the importance of carefully considering implant choice in THR for NOF fractures, particularly in elderly patients with compromised bone quality. Cemented THRs appear to offer better outcomes in this demographic, potentially due to their lower revision rates and fewer complications.

Head size also plays a crucial role in outcomes. Larger head sizes (≥32 mm) demonstrated better results in terms of reduced revision rates, as corroborated by the Australian Joint Registry [16]. Larger heads provide greater stability, reducing the risk of dislocation, a major concern in THR for NOF fractures. In contrast, smaller head sizes (<28 mm) have been associated with higher rates of dislocation and early implant failure, trends supported by registry data [16]. The greater stability offered by larger head sizes is especially important in the elderly population, where muscle strength and coordination may be compromised, increasing the risk of implant instability.

Dual mobility cups, which combine a small femoral head within a large polyethylene liner, are designed to enhance stability and reduce dislocation risk. In our cohort, no dislocations occurred in patients with dual mobility implants, supporting their efficacy in high-risk patients. Recent literature continues to support their use, with a 2024 systematic review showing reduced dislocation and overall revision rates compared to conventional total hip arthroplasty [17]. However, conflicting data from international registry analyses, including the Swedish and Dutch Arthroplasty Register, and an international meta-analysis of joint registers indicate no clear reduction in all-cause revision risk and suggest a potential increase in infection-related revisions [14,18,19]. These findings echo concerns previously raised in the 2023 Australian registry report regarding elevated revision rates in NOF cases [16]. These failures may be linked to liner wear, detachment, or infection over time, emphasizing the need for careful patient selection and long-term monitoring when using dual mobility implants.

Further addressing implant stability, posteriorly augmented liners have emerged as a viable option, particularly beneficial in cases of posterior soft tissue deficiency or compromised anatomy. These liners offer enhanced stability over standard designs without the mechanical constraints associated with fully constrained liners, representing a balanced approach in managing potential dislocation risks. In our cohort, the incorporation of posteriorly augmented liners could be explored as a strategy to further improve implant stability, particularly in patients identified as high risk for dislocation.

The functional benefits of THR in NOF fractures are well-established. Patients undergoing THR generally experience better hip function, greater mobility, and improved quality of life compared to those receiving hemiarthroplasty or internal fixation. The average Oxford Hip Score of 39.8/48 in our cohort supports this, indicating good functional outcomes in most patients. Early mobilization is crucial to achieving these positive outcomes. Studies have consistently shown that early weight-bearing and mobilization following THR significantly reduce complications such as deep vein thrombosis, pressure ulcers, and pneumonia [20]. Moreover, early mobilization has been linked to improved functional recovery and shorter hospital stays. Evidence from a systematic review and meta-analysis demonstrated that early mobilization post-THR, within 24-48 hours, resulted in faster recovery of mobility and lower postoperative complication rates [21]. Encouraging early mobilization should, therefore, be prioritized in post-THR care for patients with NOF fractures, especially in the elderly, to optimize recovery and reduce the risks of immobility.

Finally, while THR may offer functional benefits, the increased mortality in patients treated with THR for NOF fractures compared to those undergoing THR for osteoarthritis underscores the importance of careful patient selection. Frail or medically compromised individuals may not tolerate the demands of THR as well, and alternative treatments such as hemiarthroplasty may be more appropriate in these cases [22]. Ultimately, the decision to proceed with THR should be based on a comprehensive assessment of the patient’s mobility, cognitive function, and overall medical fitness, with a clear focus on balancing the benefits of improved function with the risks of surgical complications and mortality.

However, there are limitations to this study. The retrospective, single-center nature of this study increases the risk of selection and information bias. We tried to limit this by standardizing data collection through the use of the National Hip Fracture Database, conducting an internal audit of the data to ensure accuracy, and using a widely used, validated patient-reported outcome measure in the form of the Oxford Hip Score to assess functional outcomes. As with any self-reported survey, there is also a risk of reporter and response bias, particularly as the Oxford Hip Score was only collected for patients surviving beyond one year, potentially excluding less healthy individuals. Furthermore, the lack of long-term radiographic follow-up to assess implant performance, such as the incidence of loosening or wear, limits the objective evaluation of implant longevity. Finally, variability in surgical technique and implant preference between surgeons may confound the data. Future studies should involve a multicenter prospective design or meta-analysis of multiple single-center studies, comparing results with national joint registries to further enhance generalizability across diverse patient populations and clinical settings. Comparing the data to hemiarthroplasty patients would also provide a broader view of favorable methods of approach to NOF fractures.

## Conclusions

This study highlights the importance of an evidence-based approach in managing NOF fractures in elderly patients undergoing THR. Based on our findings, the use of cemented stems and larger diameter heads of 32 mm or more is recommended to reduce complications such as dislocation and periprosthetic fractures. While dual mobility cups can reduce the risk of dislocation, their use should be justified, as registry data indicate higher failure rates in NOF fractures. Early mobilization is critical to improving patient outcomes and should be a key component of postoperative care. To optimize outcomes and reduce mortality, careful patient selection based on cognitive function, mobility, and overall fitness is essential. Future research should focus on further refining implant choice and surgical timing to minimize complications.
